# Etiological Treatment of Cardiac Amyloidosis: Standard of Care and Future Directions

**DOI:** 10.1007/s11897-025-00701-4

**Published:** 2025-04-15

**Authors:** Yu Fu Ferrari Chen, Alberto Aimo, Vincenzo Castiglione, Olena Chubuchna, Paolo Morfino, Iacopo Fabiani, Gabriele Buda, Michele Emdin, Giuseppe Vergaro

**Affiliations:** 1 Health Science Interdisciplinary Center, Scuola Superiore Sant’Anna, Pisa, Italy; 2 Division of Cardiology and Cardiovascular Medicine, Fondazione Toscana Gabriele Monasterio, Pisa, Italy; 3 Department of Experimental and Clinical Medicine, University of Pisa, Pisa, Italy; 4 Health Science Interdisciplinary Center, Scuola Superiore Sant’Anna Fondazione Toscana Gabriele Monasterio, via G. Moruzzi 1, Pisa, 56124 Italy

**Keywords:** Treatment, Cardiac amyloidosis, Therapy, Stabilizer, Silencer, Chemotherapy, Tafamidis, Acoramidis, Monoclonal antibodies, Gene editing

## Abstract

**Purpose of Review:**

Cardiac amyloidosis (CA) is a condition caused by interstitial infiltration of misfolded proteins structured into amyloid fibrils. Transthyretin (ATTR) and immunoglobulin light chain (AL) amyloidosis represent the most common forms of CA. CA was traditionally perceived as a rare and incurable disease, but diagnostic and therapeutic advances have undermined the conventional paradigm.

**Recent Findings:**

The standard of care for ATTR-CA include agents capable of selectively stabilizing the precursor protein (e.g., tafamidis), whereas the plasma cell clone is the main target of chemotherapy for AL-CA. For long, tafamidis represented the only drug approved for patients with ATTR-CA. Recent data from ATTRibute-CM led to the approval of acoramidis, whereas patisiran received refusal based on the APOLLO-B trial. Novel CRISPR-Cas9-based drugs (i.e., NTLA-2001) hold great potential in the setting of ATTR-CA. Several hematological regimens are available to treat AL-CA. The main limit of current therapies is their inability to trigger removal of amyloid from tissues. However, the investigation of monoclonal antibodies targeting misfolded ATTR (e.g., PRX004, NI301A) or AL (e.g., birtamimab, anselamimab) has led to encouraging results.

**Summary:**

Various cutting-edge strategies are being tested for treatment of CA and may change the prognostic landscape of this condition in the next years.

## Introduction

Amyloidosis includes a heterogeneous category of diseases in which autologous proteins undergo misfolding and aggregation into amyloid fibrillary structures, which deposit in the extracellular space of various tissues [1]. The presence of amyloid substance induces a progressive organ damage, mediated by either disruption of tissue architecture or direct cytotoxic effects [2, 3]. The natural history of amyloidosis depends on the main sites of amyloid deposits. Myocardial amyloid infiltration progressively leads to a stiffened and pseudo-hypertrophic myocardium, which is characterized by diastolic dysfunction with restrictive pattern and then impaired systolic function [2, 4]. Cardiac involvement is the main determinant of poor prognosis [1].

Most cases of CA are caused by the accumulation of transthyretin (TTR) or immunoglobulin free light chains (FLC). TTR is a tetrameric protein largely synthetized by liver that acts as a carrier of thyroid hormones and retinol binding protein [5]. TTR amyloidosis (ATTR) can be classified into acquired (wild type [ATTRwt]) or hereditary (variant [ATTRv]) based on the presence of genetic mutations in the *TTR* gene. Light chains amyloidosis (AL) is a primarily hematologic disorder associated with abnormal plasma cells clones resulting in the presence of a monoclonal component [5, 6].

**Fig. 1:**
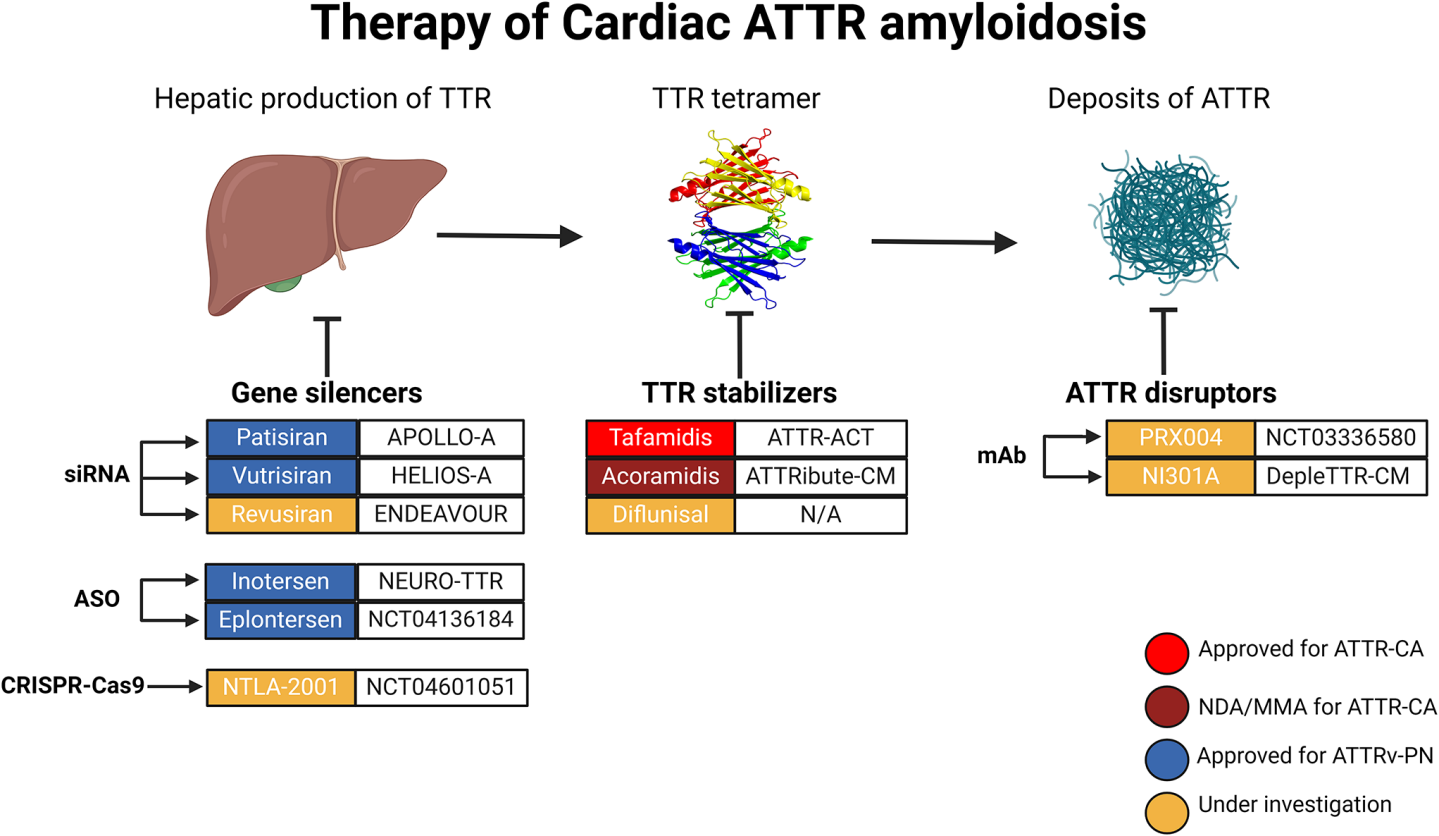
Treatment options for cardiac transthyretin amyloidosis. ASO, antisense oligonucleotides, ATTR, transthyretin amyloidosis; CA, ATTRv-PN, hereditary ATTR with polyneuropathy; cardiac amyloidosis; mAb, monoclonal antibodies; MMA, marketing authorization application; NDA, new drug application; N/A, not applicable; siRNA, small interfering RNA; TTR, transthyretin

**Fig. 2:**
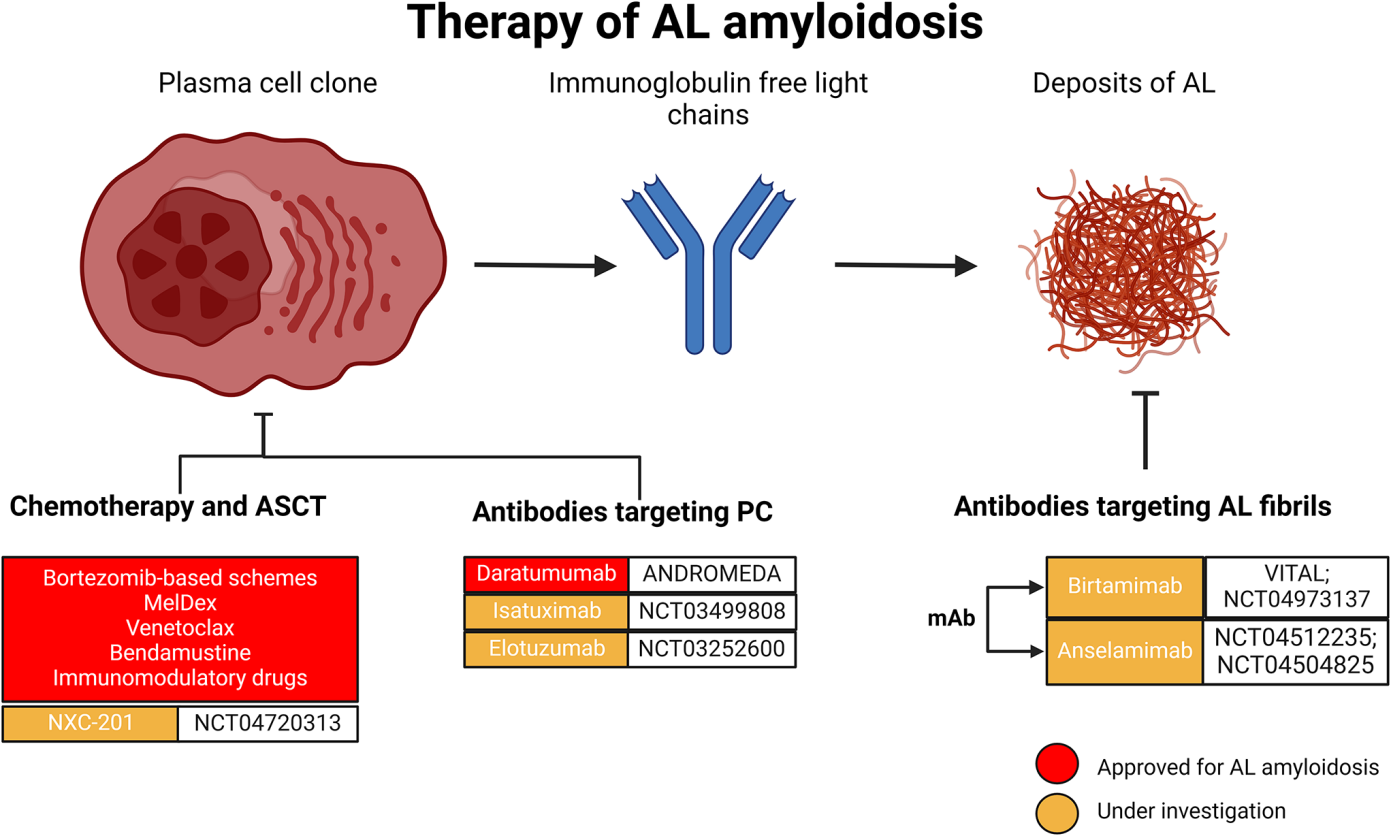
Treatment options for light-chain (AL) amyloidosis. ASCT, autologous stem cell transplant; mAb, monoclonal antibodies; MelDex, melphalan-dexamethasone; PC, plasma cell clone

The development of novel disease-modifying therapies has changed the paradigm of CA, which was traditionally perceived as an uncurable disease. In ATTR amyloidosis, the standard of care includes agents capable of stabilizing or knocking down liver synthesis of TTR, while treatment of AL amyloidosis is based on chemotherapy targeting the plasma cell clone [7, 8]. Despite being associated with improved outcomes and beneficial effects, therapies for CA limit disease progression, but are not generally able to induce amyloid regression. Novel strategies, such as selective antibodies, are being tested in clinical trials, and the therapeutic options will probably expand in the next years (Table 1) [9]. This review aims to summarize currently available therapies for CA and to highlight recent advances in the treatment of ATTR- (Fig. 1) and AL-CA (Fig. 2).

**Table 1:** Therapeutic strategies for transthyretin amyloidosis. ASO, antisense oligonucleotide; ATTR, transthyretin amyloidosis; ATTRv-PN, hereditary ATTRv with polyneuropathy; CA, cardiac amyloidosis; CO, cardiac output; CV, cardiovascular; ECV, extracellular volume; GLS, global longitudinal strain; KCCQ-OS, Kansas City cardiomyopathy Questionnaire-Overall summary; LV, left ventricular; MMA, marketing authorization application; mNIS + 7, modified neuropathy impairment score + 7, N/A, not applicable; NDA, new drug application; Norfolk QOL-DN, Norfolk quality of life diabetic neuropathy; NT-proBNP, N-terminal pro-B-type natriuretic peptide; SiRNA, short interfering RNA; SV, stroke volume; TTR, transthyretin; 6MWT, six-minute walking test

Drug name	Mechanism of action	Trial	Clinical effects	Administration	Status
Tafamidis	Tetramer stabilization	ATTR-ACT	↓ Mortality ↓ CV hospitalisation ↑ KCCQ-OS ↑6MWT ↓ NT-proBNP ↑ GLS, SV, E/e’	Oral	Approved for ATTR-CA
Acoramidis	Tetramer stabilization	ATTRibute-CM	↓ Mortality ↓ CV hospitalisation ↓ NT-proBNP ↑6MWT ↑ KCCQ-OS	Oral	Under NDA/MMA for ATTR-ACT
Diflunisal	Tetramer stabilization	N/A	Unclear	Oral	Off-label for ATTR
Patisiran	Gene silencer (siRNA)	APOLLO-B APOLLO-A	↑6MWT ↑ KCCQ-OS ↓ NT-proBNP ↓LV mass, ↑ GLS, SV ↓ECV ↓ mNIS + 7 ↓ Norfolk QOL-DN	Intravenous (once every 3 weeks)	Approved for ATTRv-PN Under investigation for ATTR-CA
Vutrisiran	Gene silencer (siRNA)	HELIOS-A and HELIOS-B	↓ NT-proBNP ↑ CO, SV ↓ mNIS + 7 ↓ Norfolk QOL-DN	Subcutaneous (once every 3 months)	Approved for ATTRv-PN Under investigation for ATTR-CA
Inotersen	Gene silencer (ASO)	NEURO-TTR	↓ mNIS + 7 ↓ Norfolk QOL-DN	Subcutaneous (weekly)	Approved for ATTRv-PN
Eplontersen	Gene silencer (ASO)	CARDIO-TTRansform and NEURO-TTRansform	↓Myocardial uptake ↓ mNIS + 7 ↓ Norfolk QOL-DN	Subcutaneous (once every 4 weeks)	Approved for ATTRv-PN Under investigation for ATTR-CA
NTLA-2001	Gene editing (CRISPR-Cas9)	MAGNITUDE	↓↓↓ serum TTR	Intravenous	Under investigation for ATTR
PRX004	Monoclonal antibody	NCT05442047	↑GLS ↓ NIS	Intravenous (once every 3 weeks)	Under investigation for ATTR-CA
NI006	Monoclonal antibody	Deple-TTR	↓Myocardial uptake ↓ECV ↓ NT-proBNP ↑ KCCQ-OS	Intravenous (once every 4 weeks)	Under investigation for ATTR-CA

## ATTR Amyloidosis

### TTR Stabilizers

The first approved treatments for patients with ATTR amyloidosis and isolated cardiac involvement is the TTR stabilizers tafamidis. The mechanism of action of stabilizing agents consists of binding physiological sites or mimicking stabilizing *TTR* variants to prevent the dissociation of TTR tetramers [10]. Stabilizers may slow down disease progression and might be particularly effective in early disease stages.

#### Tafamidis

Tafamidis stabilizes serum TTR by occupying the T_4_-binding sites [11]. It can be orally administered and is generally safe and well tolerated [12]. Tafamidis was initially recommended for the treatment of early ATTRv with polyneuropathy (ATTR-PN). In 2019, tafamidis became the first disease-modifying agent approved by Food and Drug Administration (FDA) for ATTR-CA [13–15].

The phase 3 ATTR-ACT trial, which enrolled 441 patients with ATTR-CA, explored the efficacy of tafamidis compared to placebo [13]. Patients receiving tafamidis showed a 30% reduction of all-cause mortality (hazard ratio [HR] 0.70, 95% confidence interval [CI] 0.51–0.96) and cardiovascular (CV) hospitalizations (relative risk ratio 0.68, 95% CI 0.56–0.81) over a median of 30 months. Moreover, tafamidis was capable of halting functional status decline, as assessed by 6-minute walking test (6MWT) and indirect measures of quality of life (i.e., Kansas City Cardiomyopathy Questionnaire-Overall Summary [KCCQ-OS]). A period of about 18 months was needed before survival curves for all-cause death started to diverge [13]. Patients belonging to the New York Heart Association (NYHA) functional class III displayed no benefit from tafamidis in terms of mortality and CV-related hospitalizations [13]. After a median follow-up of nearly 60 months in the open-label extension phase, tafamidis proved effective in reducing mortality even in NYHA class III, although it was less effective than in NYHA I or II patients [16, 17]. An exploratory analysis of ATTR-ACT cohort revealed that tafamidis attenuated the decline of both systolic (left ventricular [LV] stroke volume, global longitudinal strain [GLS]) and diastolic (septal and lateral E/e′) echocardiographic indices compared with placebo users [18]. A recent *post-hoc* analysis of ATTR-ACT investigated tafamidis therapy among octogenarians. After 30 months, tafamidis significantly attenuated the decline of functional capacity and quality of life (i.e., 6MWT and KCCQ-OS) and the rise of N-terminal pro-B-type natriuretic peptide (NT-proBNP) levels in patients aged $$\:\ge\:$$80 years compared to placebo, without affecting the rate of CV hospitalizations. In the open-label extension phase, tafamidis showed a trend toward reduced all-cause mortality in octogenarians at 60 months, with a median survival of 45 vs. 27 months in continuous tafamidis and placebo to tafamidis arms, respectively [19]. A major concern of tafamidis is its cost-effectiveness profile. Based on US registers, the current list price of tafamidis (approximately $250,000/year) exceeds the traditional thresholds of cost-effectiveness, and a >90% reduction of price would be needed to consider tafamidis cost-effective [20].

#### Acoramidis

Acoramidis (AG10) is a selective TTR stabilizer, which binds the tetramer with greater affinity compared to tafamidis or diflunisal in vitro. AG10 mimics the effects of the *T119M* variant, which naturally stabilizes the tetramer by inducing the formation of hydrogen bonds within the 117-serine residues [21]. In a phase 2 trial enrolling 49 patients with ATTR-CA, AG10 therapy for 28 days was well tolerated and capable to enhance circulating TTR levels, which is an indirect proof of TTR stabilization in vivo [22].

The ATTRibute-CM trial is a phase 3 multi-centre trial that investigated the efficacy of acoramidis among 632 patients with ATTR-CA for a median follow-up of 30 months [23]. Participants were randomized to acoramidis or placebo in a 2:1 ratio. After 12 months, acoramidis did not cause any improvement in exercise capacity compared to placebo, and a similar decline in 6MWT was detected [24]. However, patients treated with acoramidis reported improved KCCQ-OS measures and a significant decrease of serum NT-proBNP, along with increased concentrations of serum TTR [24]. The encouraging results of ATTRibute-CM trial were presented at the 2023 European Society of Cardiology Congress [25]. When comparing acoramidis and placebo arms, the primary endpoint (hierarchical clustering of all-cause mortality, CV hospitalization, change of serum NT-proBNP levels, and change in 6MWT) was significantly improved among acoramidis users (win ratio 1.77, 95% CI 1.42–2.22) [26]. Patients receiving acoramidis also showed reduced all-cause mortality (HR 0.77, 95% CI 0.54–1.10), cumulative frequency of CV-related hospitalisations (relative risk reduction 50.4%, 95% CI 30.5–64.5%) less pronounced increase of serum NT-proBNP and 6MWT measures from baseline [25, 26]. These results led to FDA approval of acoramidis for both ATTRwt-CA and ATTRv-CA in November 2024 [27], followed by a recommendation for approval by the EMA in December 2024 [28].

The inclusion criteria for the ATTRibute-CM trial were similar to those of ATTR-ACT. However, inclusion criteria in ATTRibute-CM required lower NT-proBNP (300 vs. 600 ng/L) and 6MWT (150 vs. 100 m) measures. The baseline characteristics of patients slightly differed across the two trials. Although acoramidis-treated patients in ATTRibute-CM were older than tafamidis-treated patients in ATTR-ACT, they had a better health status, with a larger prevalence of NYHA I-II class. Moreover, nearly 20% of patients enrolled in the ATTRibute-CM received tafamidis from the 12th months of follow-up, thus improving prognosis and reducing mortality or CV events in both acoramidis and placebo arms. As a result, the placebo group of ATTRibute-CM showed a decreased mortality after 30 months when compared with the tafamidis group of ATTR-ACT (74.3% vs. 70.5%) [13, 26].

#### Diflunisal

Diflunisal is a non-steroidal anti-inflammatory agent with stabilizing effect on TTR tetramer in vitro and in human serum [29]. A phase 2/3 trial enrolling patients with ATTRv-PN reported some beneficial effects on neurological function [30]. In the context of ATTR-CA, evidence about the efficacy of diflunisal on cardiac function and prognosis is scarce and need to be furtherly evaluated in dedicated randomized controlled trials [31–33]. The permanent block of cyclo-oxygenases could also result in renal or gastrointestinal toxicities. Therefore, the chronic use of diflunisal may be contraindicated in case of fluid overload or arterial hypertension.

### TTR Gene Silencers

TTR gene silencers include molecules capable of knocking down the hepatic expression of TTR [34]. Small interfering RNAs (siRNA) and antisense oligonucleotides (ASO) represent the main drug classes belonging to TTR silencers and induce the TTR-mRNA disruption in the liver with different mechanisms. Both siRNA (e.g., patisiran, vutrisiran) and ASO (e.g., inotersen, eplontersen) received approval for treatment of ATTRv-PN. However, no TTR silencer has been approved for ATTR-CA yet. The recent results of APOLLO-B trial, which is the unique trial assessing a gene silencer in ATTR-CA with published results to date, could impact on the future use of patisiran [35].

#### Patisiran

Patisiran is a siRNA enveloped by a lipid nanoparticle that knocks out TTR synthesis within the hepatocytes by activating the argonaute slicer protein, which then cleaves the TTR-mRNA [36, 37]. Patisiran represents the first siRNA specifically developed for ATTR amyloidosis, and was approved by the FDA for treatment of ATTRv-PN in 2018 [38].

In the phase 3 APOLLO trial, enrolling 255 ATTRv-PN, intravenous patisiran at a single dose every 3 weeks for 18 months was associated with neurological improvement and quality of life [39, 40]. Among 126 patients with ATTRv and cardiac involvement at echocardiogram, patisiran was able to induce beneficial cardiac remodelling (reduction of LV wall thickness, increase in LV end-diastolic volume, and change in GLS), reduction of NT-proBNP levels and increase in 6MWT when compared to placebo [39]. A recent case series including 16 patients with ATTRv-CA treated with patisiran reported amyloid regression, which was defined by a decrease in extracellular volume (ECV) measured by cardiac magnetic resonance (CMR), in approximately 80% of subjects treated for 12 months [41]. Of note, 12 of them received patisiran in addition to diflunisal.

The efficacy of patisiran in ATTR-CA has been assessed in the recent phase 3 APOLLO-B trial, which enrolled 360 patients with ATTR-CA followed up for 12 months [35]. Patients on patisiran showed reduced decline of functional status (i.e., 6MWT) and quality of life (i.e., KCCQ-OS) compared to placebo arm. From baseline assessment, patisiran was associated with beneficial effects on functional and structural echocardiographic parameters (LV mass, GLS, and stroke volume). Despite being positive for the primary endpoint (change in 6MWT), APOLLO-B showed that patisiran was not superior to placebo in improving prognosis of ATTR-CA patients: no significant changes in the composite endpoint of all-cause mortality, CV events, and change of 6MWT (win ratio 1.27, 95% CI 0.99–1.61) or in the composite endpoint of all-cause mortality, all-cause hospitalization and urgent visits for HF (HR 0.88, 95% CI 0.58–1.34) were observed [35]. Such results of APOLLO-B trial could be due to the duration of follow-up, which was relatively short if compared to that of ATTR-ACT or ATTRibute-CM trials. The FDA denied the approval of patisiran for patients with ATTR-CA, assessing that “the clinical meaningfulness of patisiran for this indication had not been established”, although they did not identify any issues in terms of clinical safety, study protocol, drug quality or manufacturing [42].

At the end of the 12-months double-blind phase, patients who completed the follow-up were recruited in the open-label extension phase. Therefore, novel long-term results are expected. Primary results at 18 and 24 months suggested that patisiran is still effective in preserving functional capacity and quality of life (i.e., 6MWT and KCCQ-OS) and in attenuating NT-proBNP elevation [43, 44]. Subjects switching to patisiran showed a stabilization of their clinical parameters similar to those originally included in the patisiran arm. Although the study was not properly powered, the secondary endpoints (all-cause mortality, hospitalization, and urgent HF visits) showed a trend towards improved outcomes which is worthy of being furtherly analysed [44].

#### Vutrisiran

Vutrisiran is a second-generation siRNA conjugated to N-acetylgalactosamine which can be efficiently administered subcutaneously [34]. Therefore, vutrisiran can be regularly administered with prolonged dosing intervals and without premedication with corticosteroid, acetaminophen, H1 and H2 blockers compared to patisiran (i.e., every 3 months vs. 3 weeks). Vutrisiran has been approved in 2022 for ATTRv-PN by FDA [38]. A phase 1 trial conducted on healthy volunteers reported that vutrisiran is rapidly absorbed, with a single subcutaneous infusion (25 mg) capable of inducing a serum TTR decrease of 80%, lasting for 90 days [45].

The phase 3 HELIOS-A trial, which enrolled 164 patients with ATTRv-PN, randomized participants to vutrisiran or patisiran in a 3:1 ratio and compared clinical endpoints to the placebo arm from the APOLLO trial [46]. After 18 months, vutrisiran was associated with a significant improvement in neurological function (assessed by modified neuropathy impairment score + 7 [mNIS + 7] and Norfolk Quality of Life Diabetic Neuropathy [QOL-DN] scores) compared to placebo [46]. A recent *post-hoc* analysis of HELIOS-A confirmed that vutrisiran and patisiran exert similar effects on the neurological manifestations of ATTRv amyloidosis [47]. A recent exploratory analysis enrolled patients with cardiac involvement from HELIOS-A trial and placebo arm of APOLLO-A. Treatment with vutrisiran was associated with NT-proBNP levels lower than placebo group. Additionally, vutrisiran led to an improvement of echocardiographic parameters (cardiac output, stroke volume) and a reduction of myocardial uptake on scintigraphy with bone tracers from baseline [48].

The phase 3 HELIOS-B study explored the effects of vutrisiran among patients with ATTR-CA at 30–36 months: vutrisiran reduced the risk of death for any cause (HR in overall population 0.72, 95% CI 0.56 to 0.93; *p* = 0.01; HR in the monotherapy population 0.67; 95% CI 0.49 to 0.93; *p* = 0.02) and cardiovascular events than placebo and maintained functional capacity (6MWT least squares mean difference, 26.5 m; *p* < 0.001) and quality of life (KCCQ-OS score least squares mean difference, 5.8 points; *p* < 0.001) [49]. Therefore, the study is the first to provide data about the combination therapy of TTR stabilizers and gene silencers.

#### Inotersen

Inotersen is a 2 ´-O-methoxyethyl-modified ASO that triggers ribonuclease H1-mediated disruption of the TTR mRNA. Inotersen represents the first ASO developed and approved by FDA for treatment of ATTRv-PN in 2018 [34]. Inotersen is administered subcutaneously with a weekly dose of 300 mg in Europe and 284 mg in the US.

The phase 3 NEURO-TTR trial was designed to investigate the effects of inotersen in 172 patients with ATTRv-PN followed up for 15 months [50]. Patients receiving inotersen showed a significantly lower decline of neurological function (assessed by mNIS + 7 and Norfolk QOL-DN scores) compared to the placebo group. However, inotersen was discontinued in 14% of patients because of adverse events, mostly related to injection reactions. Thrombocytopenia (defined as platelet count < 140,000/mm^3^) was detected in > 50% of patients and one patient died due to intracranial hemorrhage, for this reason patients should undergo regular platelet count checks. Severe glomerulonephritis was registered in 3 patients on inotersen, for this reason monitoring renal function every 2 weeks is recommended [50]. A recent *post-hoc* analysis of NEURO-TTR trial further confirmed that inotersen is superior to placebo in halting neurological impairment in ATTRv amyloidosis [51]. In the subgroup of patients with cardiac involvement, inotersen treatment was not associated with any beneficial effect on echocardiographic parameters (LV wall thickness, LV mass, LV ejection fraction, lateral E/e′, and GLS) after 15 months [50].

#### Eplontersen

Eplontersen is an ASO with a primary sequence homologue to inotersen, but it isconjugated to N-acetylgalactosamine [52]. In December 2023, eplontersen was approved by the FDA for the treatment of ATTRv-PN [53]. Eplontersen is estimated to be 50 times more effective than inotersen in knocking down TTR expression in hepatocytes [53, 54].

The phase 3 NEURO-TTRansform and CARDIO-TTRansform trials were designed to investigate eplontersen among patients with ATTRv-PN and ATTRv-CA, respectively. The NEURO-TTRansform trial included 168 participants treated with either eplontersen (45 mg) once every month or inotersen (300 mg) once weekly for 66 weeks [55]. An interim analysis conducted after 35 weeks was presented in September 2022, demonstrating that eplontersen efficiently slowed the progression of neuropathic disease and improved quality of life compared to an external placebo arm [56]. The final results of NEURO-TTRansform were recently published and confirmed that patients receiving eplontersen had a sustained benefit on serum TTR concentration (least squares mean reduction of 82%), neurological decline and quality of life after 66 weeks [55]. A *post-hoc* analysis of NEURO-TTRansform also suggested that eplontersen may cause a reduction of myocardial uptake on bone scintigraphy [57]. The CARDIO-TTRansform study recruited 1438 ATTR-CA patients and randomised them to eplontersen or placebo for 140 weeks. Eligible patients will also be included in a CMR sub-analysis to investigate the impact of eplontersen on indirect measures of amyloid burden (e.g., ECV) and in a sub-analysis to investigate the effects on myocardial uptake at cardiac scintigraphy with bone tracers [58].

### *TTR* Gene Editing

The clustered regularly interspaced short palindromic repeats associated Cas9 endonuclease (CRISPR-Cas9) system represents a novel and intriguing option for a series of diseases, because it offers the possibility to achieve selective in vivo gene modification [59]. ATTR amyloidosis is a monogenic disease and represents an exemplar condition for which CRISPR-Cas9-mediated gene-editing therapy could be applied. CRISPR-Cas9 system has the potential to induce a near-complete and sustained knockdown of both wild-type and variant *TTR* gene expression after a single administration [34].

#### NTLA-2001

NTLA-2001 is a CRISPR-Cas9-based genome-editing treatment, which is included and transported by a lipid nanoparticle complex to surface of hepatocytes. In preclinical mouse models, a single administration of NTLA-2001 induced a stable modification of the *TTR* gene, which was associated with a 96% reduction in serum TTR concentrations that persisted over 1 year [60]. These primary results were replicated in more complex animal models including cynomolgus monkeys and transgenic mice for human TTR-V30M mutation. No serious adverse events were detected after NTLA-2001 infusion [60].

An open-label phase 1 study conducted on patients with ATTRv-PN and ATTR-CA is investigating the safety and efficacy of NTLA-2001 [61]. An interim analysis on six patients with ATTRv-PN demonstrated that patients well tolerated NTLA-2001 infusion with limited mild adverse events [62]. After 1 month, NTLA-2001 led to a dose-dependent reduction of serum TTR concentrations, which ranged from 52% (range 47–56%) in patients receiving 0.1 mg/kg to 87% (range 80–96%) in those receiving 0.3 mg/kg. Based on CRISPR-Cas9 technology, the *TTR* knockdown induced by NTLA-2001 should be permanent and the risk of off-target gene editing needs to be cautiously managed. Computational analyses and biochemical assays estimated that the NTLA-2001 risk of off-targeting *TTR* gene is low [62].

An interim analysis on patients with cardiac involvement was presented at the 2022 American Heart Association Congress [63]. Twelve patients were included, with 10 having ATTRwt-CA and 6 in NYHA III class. Each patient reached a $\:\ge\:$90% TTR reduction at 1 month. NTLA-2001 was generally safe and well tolerated with limited mild or moderate adverse events and a single severe infusion-related reaction. After 6 months, patients in NYHA I-II class receiving NTLA-2001 0.7 mg/kg (*n* = 3) showed a sustained response with a mean 93% reduction in serum TTR levels. In the NYHA III receiving 0.7 mg/kg (*n* = 3) and NYHA I-II receiving 1.0 mg/kg (*n* = 6) groups, mean reductions of 94% and 92% respectively were reported after 4 months. The dose expansion phase of the study is ongoing, and future results will elucidate the long-term safety of NTLA-2001 [63].

The phase 3 MAGNITUDE trialhas started with dosing of first patient and it will explore the effects of NTLA-2001 compared to placebo in around 765 patients with ATTR-CA [64, 65].

### Seeding Inhibitors

Amyloid formation and deposition occurs through a complex and not completely elucidated kinetics, which can be graphically represented by an S-shaped or sigmoid curve [66]. The first phase of the process (“nucleation phase”) consists of slow generation of small pre-fibrillary aggregates (i.e., nuclei), which are then elongated by the addition of misfolded proteins until reaching thermodynamical stabilization. In vitro studies confirmed that the initial phase can be enormously shortened by pre-formed amyloid nuclei (i.e., amyloid seeds) [67]. Based on this evidence, novel molecules targeting pre-existing amyloid seeds within tissues could be effective in preventing seeding-mediated amyloidogenesis. TabFH2 is a compound tested in vitro and in *Drosophila* models of ATTR amyloidosis which was able to block amyloid formation and improve neurological function by binding the TTR sites responsible for seed formation [68].

### Monoclonal Antibodies in ATTR Amyloidosis

Monoclonal antibodies (mAb) represent a novel option for treatment of both ATTR and AL amyloidosis [9, 69]. Indeed, mAb offer the possibility to achieve an immune-mediated clearance of amyloid substance persisting within tissues through several immunological mechanisms, including complement-dependent cytotoxicity, antibody-dependent cell-mediated phagocytosis, and antibody-dependent cellular toxicity [69]. Moreover, a recent clinical series of three ATTR-CA patients demonstrated that spontaneous immune-mediated clearance of cardiac amyloid deposits normally occurs [70]. Regarding ATTR amyloidosis, preliminary results from small trials are encouraging, but further larger studies will evaluate the efficacy of PRX004 and NI006.

#### PRX004

PRX004 (NNC6019-0001) is an IgG1 antibody developed to selectively target aggregated TTR [71]. A phase 1 trial (NCT03336580) planned to explore the safety and pharmacological profile of intravenous PRX004 among 36 patients with ATTRv was early interrupted due to COVID-19 pandemic [72]. At that time, only 21 participants with ATTRv were enrolled and no serious side effects or deaths were collected [73]. Exploratory results showed that PRX004 was associated with improved neurological function (mean change of neurological impairment scale: -3.33 points) in 3/7 patients with ATTRv-PN at 9 months. An increase in GLS was documented in 7 subjects with ATTRv-CA (mean change: -1.21%) at 9 months [74]. A phase 2 multi-centre trial (NCT05442047) was then designed to investigate the efficacy of intravenous PRX004 in 99 participants with ATTR-CA at 52 weeks [75]. The first results are expected in 2024.

#### NI006

NI006 (NI301A or ALXN2220) is a human antibody which binds amyloid TTR [76]. A phase 1 trial (NCT04360434) recently evaluated the safety and pharmacological properties of intravenous NI006 infusion in 40 participants with ATTR-CA [77]. NI006 was well tolerated, and no severe drug-related adverse effects were detected. Of note, patients receiving higher doses (> 10 mg/kg) of NI006 showed a reduction in semi-quantitative parameters of myocardial uptake at scintigraphy (median heart to whole body ratio: 5.7% vs. 3.8% at 4 months and 2.5% at 12 months), and decreased ECV on CMR imaging (median 59.4% vs. 49.0% at 4 months and 41.6% at 12 months), compared with the trend to increase in placebo group. These results were accompanied by significant changes in NT-proBNP, troponin T levels and KCCQ-OS score after 12 months [77].

The phase 3 DepleTTR-CM trial was planned to investigate the efficacy of NI006 on a large cohort of ATTR-CA patients treated once every 4 weeks for 24 to 48 months (NCT06183931). The recruitment has been recently started and initial data suggest good safety profile, apart from an increased risk of mild to moderate musculoskeletal events or transient elevation of serum inflammatory markers [78].

## AL Amyloidosis

### Chemotherapy and Autologous Stem Cell Transplant

Treatment for AL amyloidosis is generally adapted from multiple myeloma therapy and is managed by hematologists. Therapy targets plasma cell clones with chemotherapy or performing autologous stem cell transplant (ASCT) in eligible subjects (< 25%) [6, 79]. The majority of patients with cardiac involvement is not eligible for transplant because of their worse prognosis [80]. The gold standard of therapy for non-transplant candidates includes daratumumab plus cyclophosphamide-bortezomib-dexamethasone (CyBorD) [81]. Daratumumab is a human IgG1κ antibody targeting CD38, which is exposed on the surface of plasma cells. Daratumumab directly triggers the apoptosis of target cells. The phase 3 ANDROMEDA trial established daratumumab plus CyBorD as the recommended first-line therapy for AL amyloidosis naïve from treatment. Cardiac response (defined as NT-proBNP > 30% and > 300 ng/L reduction in subjects with NT-proBNP ≥ 650 ng/L or NYHA class ≥ 2 reduction in subjects with baseline NYHA class III-IV) was found in 53% of patients with AL-CA after 6 months [82]. A subsequent larger study confirmed the beneficial effect of daratumumab on cardiac function, with greater rate of cardiac responses in participants on daratumumab-CyBorD vs. those on CyBorD alone (42 vs. 22%) at 6 months [83]. Patients with advanced cardiac involvement (Mayo stage IIIb or IV) are not eligible to full-dose daratumumab-CyBorD, and usually receive dose-modified daratumumab-CyBorD, single agent daratumumab or dose-modified bortezomib-based schemes [81].

The goal of treatment consists of reaching complete hematological response, which is defined as the sustained reduction of plasma FLC (difference between involved and uninvolved FLC [dFLC] < 10 mg/L or involved FLC [iFLC] < 20 mg/L). Patients who do not achieve partial response (dFLC reduction > 50% from baseline) or very good partial response (dFLC < 40 mg/L) by cycle 2 or 3, respectively, should receive dose and schedule adjustment [84]. Subjects with relapsed or refractory AL should receive combinations of proteasome inhibitors, immunomodulatory drugs, monoclonal antibodies or bendamustine (in IgM-AL amyloidosis) [81].

Besides daratumumab, other antibodies directed against plasma cell clone have been developed and tested. Isatuximab is an anti-CD38 chimeric IgG1κ which acts similarly to daratumumab but targeting a different epitope [85]. A phase 1 (NCT04754945) and 2 (NCT03499808) trials are evaluating isatuximab in subjects with advanced AL and relapsed or refractory AL, respectively. Elotuzumab is another humanized IgG1κ which targets plasma cell clone by binding the SLAMF7 glycoprotein [86]. A phase 2 (NCT03252600) trial is evaluating the efficacy of elotuzumab plus lenalidomide-dexamethasone combined or not with cyclophosphamide in relapsed AL.

Over the last decades, prognosis of patients with AL amyloidosis dramatically improved and the onset of long-term CV toxicities associated with hematological treatments represent an emerging source of concern [87]. Although generally associated with high safety and tolerability, AL patients receiving chemotherapy may experience LV dysfunction, cardiac arrhythmias, and thromboembolic events of various degree [88]. Future studies should explore the long-term cardiovascular effects of these treatments and potentially identify strategies capable of exerting a cardio-protective action.

### Future Directions in AL Cardiac Amyloidosis

#### AL Amyloidosis with Advanced Cardiac Disease

The clinical management of patients with AL and advanced cardiac involvement is still suboptimal. In particular, subjects in Mayo stage IIIb (NTproBNP > 8500 ng/L or BNP > 700 ng/L, troponin T > 0.035 µg/L or troponin I > 0.1 µg/L or high-sensitivity troponin T > 54 ng/L), who accounts for around 20% of AL population, are characterised by median survival of 4–6 months [89]. Novel hematological schemes can induce a complete or very good partial hematological response, which is not necessarily associated with a significant survival benefit [90]. Cardiac response is rarely achieved but is possible, as demonstrated by a recent study including 249 patients with newly diagnosed AL-CA at Mayo stage IIIb. Patients received conventional therapy before approval of daratumumab (bortezomib-based schemes, melphalan-dexamethasone, immunomodulatory drugs or rituximab). After 3 months, only 8% of participants reported a significant cardiac response (decrease of NT-proBNP > 300 ng/L and > 30%), which was associated with a longer median survival (54 months) compared to patients without cardiac response (6 months) [91]. Therefore, novel therapeutic approaches are needed to achieve early and deep organ response among patients with advanced AL-CA. As reported below, results from clinical trials investigating immunotherapy with birtamimab and anselamimab are encouraging.

NXC-201 (HBI0101) is a novel anti B-cell maturation antigen (BCMA) chimeric antigen receptor (CAR) T cell therapy [92]. NXC-201 is being tested in patients with relapsed or refractory AL amyloidosis in a phase 1/2 trial (NCT04720313). Preliminary results on 9 patients (*n* = 7 with cardiac involvement) suggested great efficacy in terms of hematological response, with complete remission in 6/9 and at least partial response in 3/9 patients. Cytokine release syndrome was detected in 7/9 patients. After a median follow-up of 7.3 months, the safety profile of NXC-201 was acceptable and no treatment-related deaths were collected [93].

### Monoclonal Antibodies in AL Amyloidosis

The anti-fibril agents birtamimab and anselamimab could change the management of AL amyloidosis with advanced cardiac involvement, as suggested by results of the recent VITAL trial [9, 94].

#### Birtamimab

Birtamimab (NEOD001) is a IgG1 which binds misfolded AL amyloid fibrils [95]. Although birtamimab was safe in patients with previously treated AL amyloidosis, the phase 3 VITAL study, that enrolled newly diagnosed AL-CA patients, did not meet its primary endpoint (composite of all-cause mortality and CV hospitalizations) and was interrupted following an interim analysis that revealed the futility of the study [96]. However, *post-hoc* analyses reported a benefit in survival and quality of life among participants (*n* = 260) with advanced AL-CA (Mayo stage IV) treated with birtamimab plus standard therapy compared to control group (survival rate of 74% vs. 49%, at 9 months) [94]. Based on these results, the phase 3 AFFIRM-AL trial is enrolling patients with AL amyloidosis at Mayo stage IV (NCT04973137).

#### Anselamimab

The IgG1 anselamimab (CAEL-101) targets misfolded AL fibrils. Anselamimab was safe and well tolerated in patients (*n* = 27) with AL amyloidosis, with 8/12 patients with cardiac involvement manifesting beneficial cardiac response (improved GLS) after 1 month [85, 86]. Two phase III trials recruiting patients with AL amyloidosis and Mayo stage III are ongoing (NCT04512235; NCT04504825).

### Pan-Amyloid Monoclonal Antibodies

Finally, the development of pan-amyloid mAb may offer the possibility to treat various forms of systemic amyloidosis through the same compound. The first attempt consisted of antibodies targeting the serum amyloid protein (SAP), which is commonly found in various type of amyloid deposits. However, the negative results of anti-SAP antibodies triggered the development of agents directed towards other targets universally shared by amyloid fibrils [97, 98].

#### Anti-Heparan Sulfate Proteoglycans

Novel mAb targeting heparan sulfate proteoglycans, which are included within amyloid deposits, are being developed and tested [99]. The common technology of these antibodies is based on the presence of a peptide (i.e., p5) which selectively targets heparan sulfate proteoglycans present in amyloid fibrils [99, 100]. The peptide was subsequently combined with an Fc fragment to generate a compound able to trigger immune response against amyloid deposits [101].

AT-02 is humanized recombinant IgG1 which binds both amyloid TTR and FLC [102]. A phase 1 trial (NCT05521022) was planned to test the safety of intravenous AT-02. Topline data are waited for 2024. AT-03 and AT-04 were proven to trigger phagocytosis of human amyloid TTR or FLC aggregates in vitro [103, 104]. AT-04 efficiently binds to cerebral amyloid, and was then modified (AT-07) to gain greater capacity of penetration into cerebral amyloid [105]. AT-06 consists of the common peptide fused with the CAR derived from macrophages (CAR-M) [102]. AT-01 and AT-05 were developed for diagnostic purpose and actually do not show any therapeutic effect [106].

## Discussion

CA has been long considered a rare condition characterized by poor prognosis. However, many advances in therapeutic strategies have transformed the landscape of this disease [7, 8]. The standard of care for ATTR-CA actually includes stabilizing agents such as tafamidis, which is the only drug approved for this condition [13]. Recent results from the ATTRibute-CM trial encouraged and led to the approval of acoramidis, which is now a feasible alternative to tafamidis as TTR stabilizer [26]. Of note, approximately 15% of patients included in the acoramidis arm of ATTRibute-CM were also treated with tafamidis starting from the 12th month of follow-up. Although evidence regarding the combined used of TTR stabilizers is lacking, preliminary data suggest that the beneficial effects of these drugs may be synergic. However, the high cost of TTR stabilizers may limit their combined use.

On the other hand, recent results from the APOLLO-B trial discouraged the use of patisiran in patients with ATTR-CA, leading the FDA to deny the expanded approval for ATTR patients with cardiac involvement [35]. Patisiran was proved to preserve functional capacity and health status without improving prognosis. The follow-up period of the study was relatively short if compared to that of trials with tafamidis or acoramidis (12 vs. 30 months). Based on the results of ATTR-ACT trial, patients with amyloidosis may benefit of etiological therapy after many months of treatment. For this reason, long-term results from the expansion phase of APOLLO-B are crucial to better understand the impact of patisiran on prognosis of ATTR-CA patients. Indeed, the Kaplen-Meier curves for mortality, hospitalization and HF exacerbation at 24 months tended to diverge, even if not significantly [44]. Results from the HELIOS-B trial clarified the clinical efficacy of vutrisiran in ATTR patients with cardiac involvement.

Despite the promising results, therapy with gene silencers need cautious monitoring, as demonstrated by inotersen, which leads to severe hematological or renal side effects in some patients [50]. Another illustrative case of poor safety is revusiran, which was a siRNA associated with increased mortality in the phase 3 ENDEAVOUR study on ATTRv patients [107].

From a pathogenic perspective, TTR stabilizers and gene silencers act through sequential mechanisms of action. Indeed, stabilizers act further downstream than gene silencers. However, TTR-synthesis inhibition and tetramer stabilization may be complementary strategies possibly achievable in the same patient. Combination therapy may be employed in patients who do not achieve satisfying results with a single treatment and when clinicians could expect greater benefits. An optimal combination of etiological drugs might also require a stabilizer and a drug that depletes ATTR deposits, as they work on different parts of the amyloidogenic ATTR cascade.

Various cutting-edge strategies are being tested and may change the treatment paradigm of ATTR-CA in the near future. Gene editing therapy with NTLA-2001 holds great potential, because the deep and sustained suppression of TTR production may prove very effective. However, definitive and long-term results are warranted to understand both the efficacy and the safety of such an innovative approach. Finally, novel mAb were developed and tested in both ATTR and AL amyloidosis. The removal of amyloid deposits through immunotherapy may dramatically reduce the cardiac amyloid burden and relieve cardiac function of treated patients. Results from phase 3 trials are expected to validate the efficacy of mAb on major clinical outcomes.

Regarding AL amyloidosis, the clinical management of patients with advanced cardiac involvement is still suboptimal, but recent studies suggest that cardiac response is achievable with a proper early treatment, especially with novel mAb such as birtamimab, evaluated in the VITAL trial. Finally, the development of pan-amyloid antibodies potentially offers a unique therapeutic solution for various forms of systemic amyloidosis, but further research is needed considering the negative background of anti-SAP antibodies.

Any patient with CA holds specific risk factors and comorbidities, which determine a different clinical profile possibly deserving of a personalized treatment. Finding the proper therapeutic strategy represents a challenge for clinicians. For instance, patisiran might be the preferred option for a patient who is receiving anticoagulants for atrial fibrillation, since treatment with inotersen could potentially lead to thrombocytopenia. The definition of methods to monitor pharmacological response, understanding when to initiate and when to discontinue therapy, and creating various pharmacological protocols are necessary to pursue a tailored and effective treatment of CA, which is an incredibly complexdisease.

## Data Availability

No datasets were generated or analysed during the current study.
